# Multi-Frame Vibration MEMS Gyroscope Temperature Compensation Based on Combined GWO-VMD-TCN-LSTM Algorithm

**DOI:** 10.3390/mi15111379

**Published:** 2024-11-15

**Authors:** Ao Li, Ke Cui, Daren An, Xiaoyi Wang, Huiliang Cao

**Affiliations:** 1 School of Engineer, The Hong Kong University of Science and Technology, Hong Kong, China; alibf@connect.ust.hk (A.L.); xiaoyiwang@bit.edu.cn (X.W.); 2 School of Instrument and Electronics, North University of China, Taiyuan 030051, China; ck18180456109@163.com; 3 Chongqing Institute of Microelectronics and Microsystems, Beijing Institute of Technology, Chongqing 401332, China; 4 Engineering Research Center of Integrated Acousto-Opto-Electronic Microsystems, Ministry of Education of China, Beijing 100816, China

**Keywords:** MEMS gyroscope, temperature compensation, GWO-VMD denoising, TCN-LSTM model, Multi-Frame Vibration MEMS Gyroscope (DMFVMG)

## Abstract

This paper presents a temperature compensation model for the Multi-Frame Vibration MEMS Gyroscope (DMFVMG) based on Grey Wolf Optimization Variational Mode Decomposition (GWO-VMD) for denoising and a combination of the Temporal Convolutional Network (TCN) and the Long Short-Term Memory (LSTM) network for temperature drift prediction. Initially, the gyroscope output signal was denoised using GWO-VMD, retaining the useful signal components and eliminating noise. Subsequently, the denoised signal was utilized to predict temperature drift using the TCN-LSTM model. The experimental results demonstrate that the compensation model significantly enhanced the gyroscope’s performance across various temperatures, reducing the rate random wander from 102.929°/h/√Hz to 17.6903°/h/√Hz and the bias instability from 63.70°/h to 1.38°/h, with reductions of 82.81% and 97.83%, respectively. This study validates the effectiveness and superiority of the proposed temperature compensation model.

## 1. Introduction

MEMS gyroscopes play a key role in modern navigation and control systems. These devices are favored for their high reliability, low interference and energy efficiency. They measure angular velocity by accurately transmitting vibration signals in their drive and sense modes. Nevertheless, a non-negligible issue is that the accuracy of MEMS gyroscopes can be significantly degraded by fluctuations in ambient temperature, which may affect their mission-critical applications. Therefore, the development of effective temperature compensation strategies is essential to ensure the performance of these devices.

In the research of the temperature compensation of the MEMS gyroscope, scholars have carried out extensive research. The main research directions can be divided into two main categories: hardware enhancement and software algorithm optimization. Hardware enhancement methods improve the gyroscope’s adaptability to temperature changes by improving its physical structure and circuit design. For example, in [1,2], Shen et al. enhanced the hardware performance by adjusting the structure of the silicon-based materials. Although this approach can theoretically enhance performance, it is usually accompanied by a higher cost and a complex manufacturing process.

Software algorithm optimization, on the other hand, uses a computer program to adjust the output of the gyroscope to temperature changes. This typically involves two steps: signal denoising and temperature drift modeling. For signal de-noising, researchers have used a variety of techniques, including serial and parallel processing. Serial processing removes noise through a filtering algorithm and then applies temperature drift modeling for compensation. Li et al. achieved temperature compensation and noise suppression by integrating an adaptive sliding window (ASW)-based Kalman filter (KF) and a statistical calibration filter (SCF) for a dual U-beam vibrating ring-based gyroscope [3]. However, this approach may affect the stability of the static drift model. Parallel processing, on the other hand, involves separating the output signals at different scales, filtering the signals at each scale separately and finally recombining them, which better preserves the accuracy of the drift model.

In signal decomposition techniques, Empirical Modal Decomposition (EMD), Ensemble Empirical Modal Decomposition (EEMD) and Variational Modal Decomposition (VMD) are a few of the common methods [4,5,6,7]. VMD is favored for its fast convergence speed and good decomposition results [8]. VMD incorporates mechanisms to mitigate modal aliasing by regularizing the bandwidth or frequency range of modal functions, enhancing decomposition accuracy and robustness. In comparison, EMD, while adept at handling nonlinear and non-stationary signals, lacks a solid mathematical foundation, is noise-sensitive and is prone to edge effects. VMD’s mathematical optimization and parameter tuning offer superior noise resistance and aliasing suppression. By using VMD with optimization algorithms for optimal decomposition and the reconstruction of signals, researchers are able to decompose signals into noise, mixed signals and characteristic signals and classify these signals using Pearson’s correlation coefficient. Pearson’s correlation coefficient is a statistic that measures the strength of a linear relationship between two variables. Its value is between −1 and 1 and can be effectively used for signal correlation analysis [9]. Therefore, the researcher used the optimized VMD to denoise and reconstruct the signals.

After obtaining the denoised signal, it is crucial to build a highly accurate drift prediction model. This model is used to predict the output signal of the MEMS gyroscope and, ultimately, the predicted value is utilized for temperature compensation. Therefore, the accuracy of the drift prediction model largely determines the effectiveness of temperature compensation. It has been shown that the accuracy of drift prediction can be significantly improved by using deep learning methods such as Long Short-Term Memory (LSTMs) networks combined with Deep Neural Networks (DNNs). This approach effectively improves the temperature compensation performance of gyroscopes by analyzing multiple environmental factors to build a more accurate prediction model. Zhang et al. [10] proposed a hybrid algorithm for temperature compensation based on Improved Adaptive Noise Fully Integrated Empirical Mode Decomposition (ICEEMDAN), Sample Entropy, Peak Time–Frequency Filtering, Non-Dominated Sorting Genetic Algorithm II (NSGA II) and Extreme Learning Machine. Li et al. [11] developed a hybrid algorithm for temperature drift prediction in a multi-input single-output temperature model utilizing an Elman neural network. However, the algorithm exhibits a relatively slow convergence speed. To this end, we propose a model based on a time convolution network (TCN) and a Long Short-Term Memory (LSTM) network (which is inspired by the lithium battery charge state estimation method proposed by Hu et al. [12]), utilizing the TCN for extracting higher level spatial features in the multivariate and the LSTM for capturing long term dependencies from time-series data. The TCN-LSTM model is capable of learning the gyroscopic drift patterns under different temperature conditions and making highly accurate predictions, thereby improving the overall performance and reliability of the gyroscope.

## 2. Multi-Frame Vibration MEMS Gyroscope

### 2.1. DMFVMG Structure Design and Working Principle

**Figure 1:**
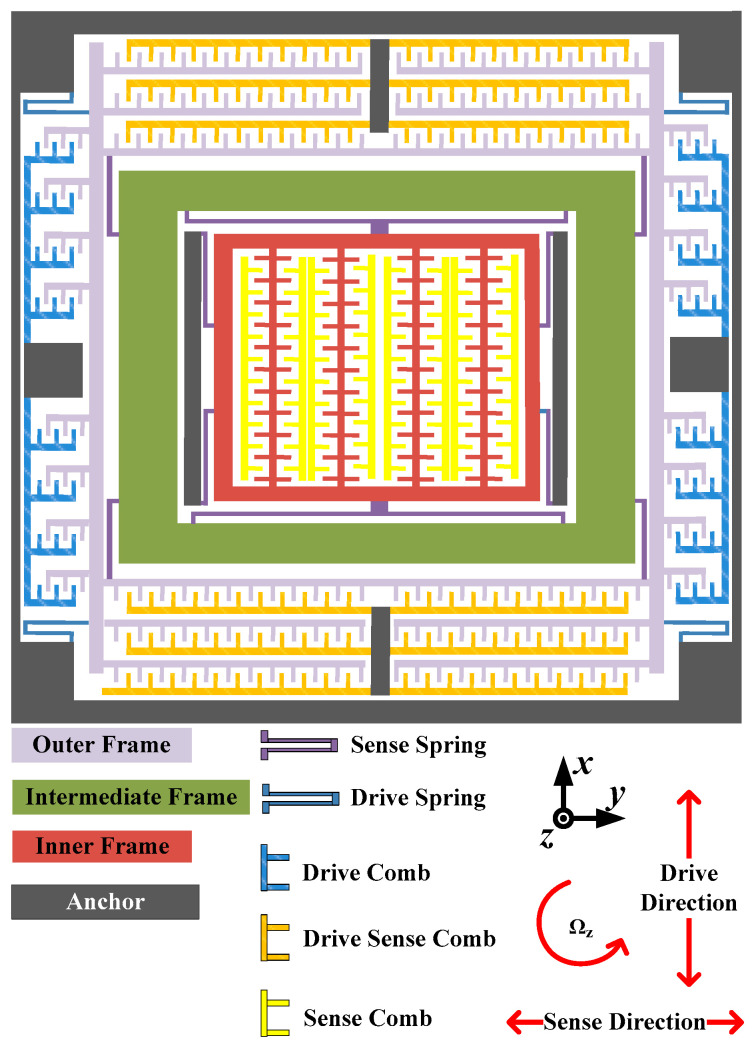
The diagrammatic representation of the DMFVMG structure.

In this paper, we utilized a Z-axis decoupled multi-frame vibrating MEMS gyroscope (DMFVMG), whose structure is depicted in Figure 1. Initially, the decoupling mechanism was employed to organize the sensing frame, Coriolis frame and driving frame through the arrangement of inner, middle and outer frames. Specifically, the outer frame served as the driving mass along the x-axis, while the inner frame functioned as the sensing mass. The middle frame, acting as a Coriolis mass block, could move in conjunction with either the outer or inner frame. The outer and middle frames were interconnected via a crab foot beam. Vertical comb capacitors were used for the drive and drive detection combs and transverse comb capacitors were used for the detection combs. The entire structure was held up by flexible beams and hung from the substrate. The outer frame, along with the middle frame, linked to it via anchor points, was supported and suspended by four angular flexible beams. When an electrostatic driving force was applied, both the outer and middle frames oscillated along the x-axis in accordance with the drive frequency. Meanwhile, the inner frame, which was connected to two anchor points and the middle frame through flexible beams [13,14], remained responsive to these movements. The operational principle of the sensor could be broken down into four stages:

1. The outer frame is actuated by electrostatic forces, causing it to move along the X-axis;
2. The intermediate frame is propelled along the X-axis by the impetus of the outer frame, aligning with the initial segment;
3. When a specific angular velocity is applied around the Z-axis, the middle frame undergoes elliptical motion in the x-o-y plane due to the combined effects of the driving force and Coriolis force;
4. The inner frame and detection comb vibrate in the y-axis direction, with the amplitude of vibration being proportional to the input angular rate.

### 2.2. DMFVMG Structure Mode Simulation

**Figure 2:**
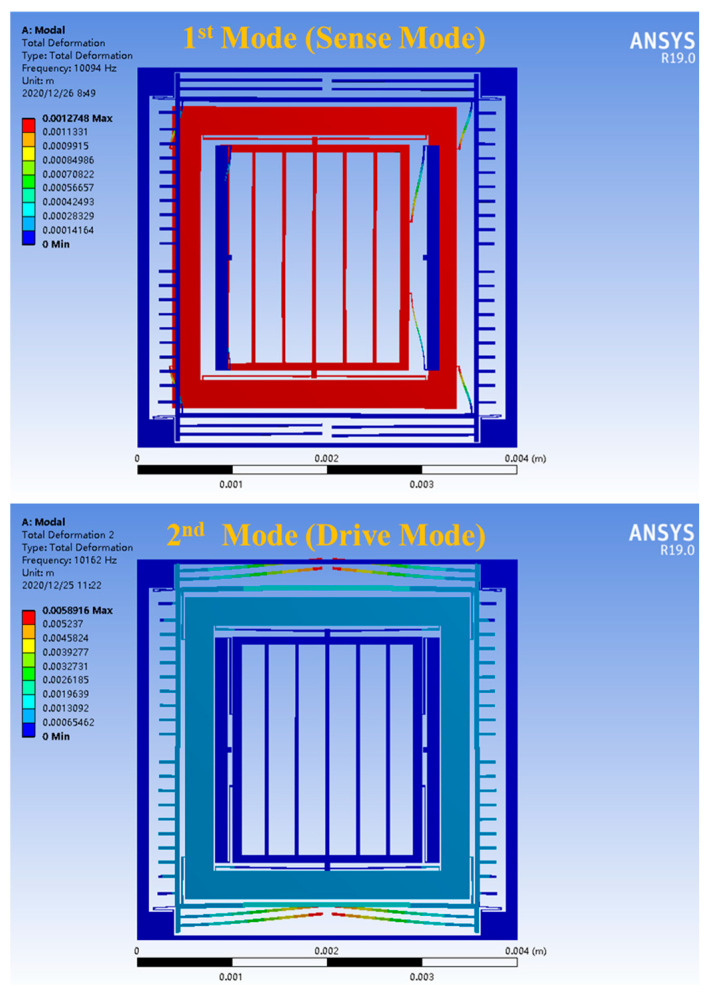
Analysis of the modal behavior of the DMFVMG structure.

Figure 2 displays the outcomes of the modeling and simulation conducted using ANSYS 19.0 (ANSYS, Inc., Southpointe, PA, USA) finite element analysis software. Specifically, it shows the induction mode (first mode) and drive mode (second mode). In the induction mode, both the inner and middle frames moved along the induction axis. Conversely, in the drive mode, the outer and middle frames moved in the drive direction, while the inner frame remained stationary. This demonstrates the effective functioning of the coupling structure. By examining Figure 2 and Table 1, it is evident that the resonance frequencies for the induction and drive modes were 10,094 Hz and 10,162 Hz, respectively, with a crossover frequency of 68 Hz. This signifies that the structure possessed high mechanical sensitivity (a lower crossover frequency corresponds to better mechanical sensitivity) and fulfilled the gyroscope’s bandwidth requirements. The frequency was sufficiently high to satisfy the gyroscope’s need for resistance to low-frequency vibrations. Furthermore, based on the frequency distribution, the crossover frequency between the lowest-order interference mode (3rd order mode at 14,986 Hz) and the working mode was 4824 Hz, indicating that the interference mode had minimal impact on the working mode [15,16].

### 2.3. Work of DMFVMG

**Figure 3:**
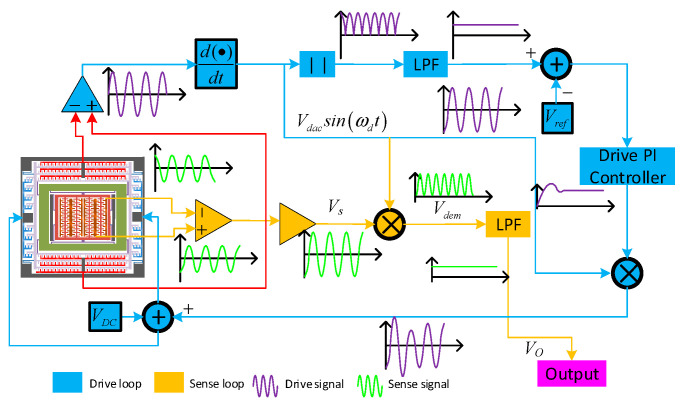
The control block diagram of DMFVMG gyro.

Figure 3 illustrates the schematic representation of the detection mechanism within a three-mass MEMS gyroscope. The system is primarily composed of two components: a drive closed-loop subsystem (enclosed in blue in Figure 3) and a sense open-loop subsystem (highlighted in yellow in Figure 3). Within the drive closed-loop subsystem, the drive signal is initially captured by the drive sensing comb. Subsequently, it is enhanced by a differential amplifier. Following amplification, the signal undergoes a phase shift equivalent to a quarter of the wavelength, facilitated by a phase delay unit, to synchronize with the drive AC signal, denoted as *V_dac_sin*(*ω_d_t*). Next, the signal undergoes envelope detection through a full-wave rectification and low-pass filtering process. Once filtered, it is fed into a voltage comparator, which then evaluates it against a predefined reference voltage, *V_ref_.* The resulting output signal is directed to the drive closed-loop PI (Proportional–Integral) controller, which in turn produces a control signal. This control signal is then modulated using the sinusoidal modulating voltage *V_dac_sin*(*ω_d_t*). Following modulation, it is combined with the DC voltage *V_DC_* and supplied to the excitation drive circuit. Within the induction open-loop subsystem, the Coriolis mass block’s motion is sensed and its signal is processed by a differential detection amplifier followed by a proportional amplifier, resulting in the signal *V_s_*. This signal *V*_s_ is then demodulated using the modulating voltage *V_dac_sin*(*ω_d_t*), yielding the demodulated signal *V_dem_*. Subsequently, *V_dem_* is filtered by a low-pass filter to generate the inductive open-loop output signal *V_o_*.

### 2.4. Temperature Effects of DMFVMG

MEMS gyroscopes, being mechanical structures, are susceptible to certain environmental factors, particularly temperature variations. Changes in temperature can considerably influence the material properties, which often exhibit a degree of sensitivity to thermal alterations. This thermal sensitivity may result in alterations to specific response physical quantities that are pivotal to the sensing process or outcomes. Hence, it is imperative to carefully consider the impact of ambient temperature on the performance and usability of DMFVMG.

The influence of temperature on the performance of MEMS gyroscopes has been addressed in the existing literature. We examined several research studies concerning gyroscopes in environments with temperature fluctuations and discovered that most researchers have adequately focused on the temperature properties of materials as the primary factor contributing to performance variations. Cao et al. examined the operational performance of MEMS gyroscopes within a temperature range of 313.15 K to 333.15 K and proposed techniques to compensate for device bias and scale factor. Their test results indicated that the scale factor is directly proportional to the resonator amplitude and accelerometer loop gain and inversely proportional to the frequency difference between the two critical operating modes. Chandradip et al. introduced a series of methods for simulating and analyzing the behavior of vibrating microgyroscopes at different temperatures, considering two key parameters: Young’s modulus and the damping factor. They presented simulation results that highlight the temperature-dependent properties of MEMS device operation. Another study offered a comprehensive analysis of MEMS gyroscope performance and the impact of temperature on their performance and usability. Xia et al. argue in their paper that as the temperature increases, the resonant frequency and quality factor of the device decrease. Fang et al. took into account the Seebeck effect and discussed the variation in Young’s modulus due to temperature changes [17,18,19,20].

## 3. Algorithms and Models

### 3.1. Variational Mode Decomposition (VMD)

Variational Mode Decomposition (VMD), introduced by K. Dragomiretskiy and colleagues, is a sophisticated adaptive signal processing technique. This method iteratively seeks the optimal solution for variational modes, simultaneously refining the mode functions and their corresponding central frequencies. Consequently, it yields several Intrinsic Mode Functions (IMFs) that encompass a specific broadband spectrum. In our study, we employed VMD to decompose the gyroscope output signal into multiple IMFs, each capturing distinct frequency bands within the signal. This approach allowed for a more nuanced analysis of the signal’s underlying components [21,22].

The essence of Variational Mode Decomposition (VMD) lies in formulating and solving a variational problem. The procedure is primarily dictated by two key limitations: on one hand, the objective is to reduce the total bandwidth of the central frequencies for each modal component and on the other hand, it is necessary to ensure that the aggregate of all the modal components reconstructs the original signal. Assuming that the original signal can be broken down into separate components, the variational formulas that include the lambda parameter constraint are detailed below:

(1) $$\left\{\begin{array}{c} \underset{\left\{u_{k}\right\}\left\{\omega_{k}\right\}}{\min}\left\{\sum_{k=1}^{K}{\left\|\left.\partial_{t}\left[(\delta (t)+\frac{j}{\pi t})*u_{k}\left(t\right)\right]e^{-j\omega_{k}t}\right\|\right.}_{2}^{2}\right\}\\ s.t.\sum_{k=1}^{K}u_{k}=f \end{array}\right.$$

Here, *K* represents the total count of the modal components that are to be separated within the decomposition process and the notation {*u_k_*(*t*)} refers to the Intrinsic Mode Function (IMF) resulting from the decomposition process, whereas *δ*(*t*) signifies the Dirac delta function.

By introducing the Lagrange multiplier, represented by *λ*, the constrained variational problem in Equation (1) can be seamlessly converted into an unconstrained problem. This transformation simplifies the optimization process and leads to the derivation of the following expressions:

(2) $$L\left(\left\{u_{k}\right\},\left\{\omega_{k}\right\},\lambda \right)=L_{1}+L_{2}$$

(3) $$L_{1}=\alpha \sum_{k=1}^{K}{\left\|\left.\partial_{t}\left\{\left(\delta (t)+\frac{j}{\pi t}\right)*u_{k}\left(t\right)\right\}e^{-j\omega_{k}t}\right\|\right.}_{2}^{2}$$

(4) $$L_{2}={\left\|\left.f(t)-\sum_{k=1}^{K}u_{k}(t)\right\|\right.}_{2}^{2}+\langle \lambda (t),f(t)-\sum_{k=1}^{K}u_{k}(t)\rangle$$

where *α* is a quadratic penalty factor to ensure the signal reconstruction accuracy.

Equation (2) is resolved through the application of the Alternating Direction Method of Multipliers (ADMM), an algorithm designed for optimization problems with coupled constraints. In the iterative solution process, the Intrinsic Mode Functions *u_k_*, the central frequencies *ω_k_* and the Lagrange multiplier *λ* are updated via Equations (5), (6) and (7), respectively.

(5) $${\hat{u}}_{k}^{n+1}(\omega )\leftarrow \frac{f(\omega )-\sum_{i=1,i<k}^{K}{\hat{u}}_{i}^{n+1}(\omega )-\sum_{i=1,i>k}^{K}{\hat{u}}_{i}^{n}(\omega )+{\hat{\lambda }}^{n}(\omega )/2}{1+2\alpha {\left(\omega -\omega_{k}^{n}\right)}^{2}}$$

(6) $$\omega_{k}^{n+1}\leftarrow \frac{\int_{0}^{\infty }\omega {\left|{\hat{u}}_{k}^{n+1}(\omega )\right|}^{2}d\omega }{\int_{0}^{\infty }{\left|{\hat{u}}_{k}^{n+1}(\omega )\right|}^{2}d\omega }$$

(7) $${\hat{\lambda }}^{n+1}(\omega )\leftarrow {\hat{\lambda }}^{n}(\omega )+\tau (\hat{f}(\omega )-\sum_{k}{\hat{u}}_{k}^{n+1}(\omega ))$$

The parameter *τ* represents the noise threshold and it influences the accuracy of the signal’s decomposition process. The symbols $\hat{f}$, $\hat{u}$ and $\hat{\lambda }$ correspond to the Fourier transforms of the variables f, u and *λ*, respectively.

The iterative process will continue until Equation (8) is satisfied.

(8) $$\sum_{k}{\left\|{\hat{u}}_{k}^{n+1}-{\hat{u}}_{k}^{n}\right\|}_{2}^{2}/{\left\|{\hat{u}}_{k}^{n}\right\|}_{2}^{2}<\varepsilon$$

The symbol *ε* is the set accuracy.

Upon the completion of the iterative process, the full set of intrinsic mode functions (IMFs) for the gyroscope’s original signal was successfully retrieved.

### 3.2. Grey Wolf Optimization Variational Mode Decomposition (GWO-VMD) Algorithm’s Digital Signal Denoising

The Grey Wolf Optimization (GWO) algorithm achieves optimization by simulating the predatory behavior of gray wolf packs, based on the mechanism of wolf pack group collaboration. The principle of the Grey Wolf Optimization algorithm is as follows:

1. In the α-tier wolf pack, the leader in the population is responsible for leading the entire wolf pack in hunting the prey, i.e., the optimal solution in the optimized algorithm;
2. In the β-tier wolf pack, responsible for assisting the α-layer wolf pack, the suboptimal solution is in the optimized algorithm;
3. δ tier wolves follow the orders and decisions of α and β and are responsible for scouting, sentry duty, etc. Poorly adapted α and β are demoted to δ;
4. ω-layer wolves update their position around α, β or δ.

GWO mimics the behavior of a wolf pack in which the leader (α), the second leader (β) and the follower (δ) search for prey and is used to solve a variety of complex optimization problems. VMD separates the different frequency components of a signal by minimizing the residual energy and is commonly used in signal processing, image analysis and other fields. When the two are combined, GWO-VMD, Grey Wolf Optimization, is used to optimize the process of VMD. Specifically, in digital signal denoising tasks, GWO can help to find the optimal combination of the VMD parameters, such as the number of fundamental frequencies, the window size, etc., in order to maximize the extraction of useful information from the signal and to reduce the effect of noise. The advantage of this approach is that it can adaptively process the signal and does not require excessive a priori knowledge of the signal characteristics.

### 3.3. TCN-LSTM Model

**Figure 4:**
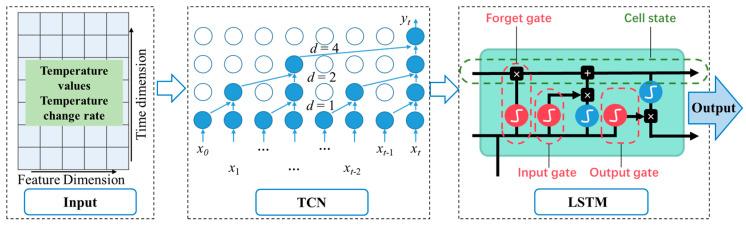
Combined gyroscope temperature drift signal prediction models.

We used a TCN-LSTM model combining Temporal Convolutional Networks (TCNs) and Long Short-Term Memory Networks (LSTMs) for time-series prediction. Figure 4 illustrates the conceptual framework of the integrated gyroscope temperature drift signal forecasting model. The subsequent steps outline the process of developing and refining this model.

1. First, we used a TCN layer to extract the features from the time-series data. The TCN processed the data through a series of convolutional layers, each of which captured patterns over different time scales;
2. Next, we took the output of the TCN as the input to the LSTM layer and utilized the recursive nature of the LSTM layer to capture long-term dependencies;
3. When training the TCN-LSTM model, we needed to choose the appropriate network structure and hyperparameters. This included determining the number of layers and the number of units per layer for the TCN layer and the number of hidden units for the LSTM layer. We could evaluate the performance of the model under different configurations and chose the best model structure through methods such as cross-validation;
4. Ultimately, we input the temperature data of the gyroscope that required compensation into the TCN-LSTM model for evaluation, thereby deriving the anticipated output signal of the MEMS gyroscope [23,24].

#### 3.3.1. Temporal Convolutional Networks (TCNs)

The TCN is a deep learning model for sequence prediction that processes time-series data by using a one-dimensional convolutional layer. The TCN is particularly well-suited for capturing long-term dependencies because it utilizes dilation convolution (also known as null convolution) to increase the receptive field of the convolutional layer without adding additional parameters. This structure allows TCNs to process data efficiently in parallel and avoids the problem of gradient vanishing found in traditional recurrent neural networks. Another key feature of TCNs is causal convolution, which ensures that the model’s outputs are only dependent on previous time steps, fulfilling the causality requirement in time-series analysis [25,26].

#### 3.3.2. Long Short-Term Memory (LSTM)

LSTM is an advanced recurrent neural network (RNN) that solves the gradient vanishing problem of traditional RNNs when dealing with long sequences by introducing a complex gating mechanism. LSTM contains an input gate, a forgetting gate and an output gate, which control the flow of information, allowing the network to keep or forget information in a cellular state. This design allows the LSTM to capture long-term dependencies in time-series data while ignoring unimportant information.

### 3.4. Compensation Model Based on GWO-VMD Denoising and TCN-LSTM Prediction

**Figure 5:**
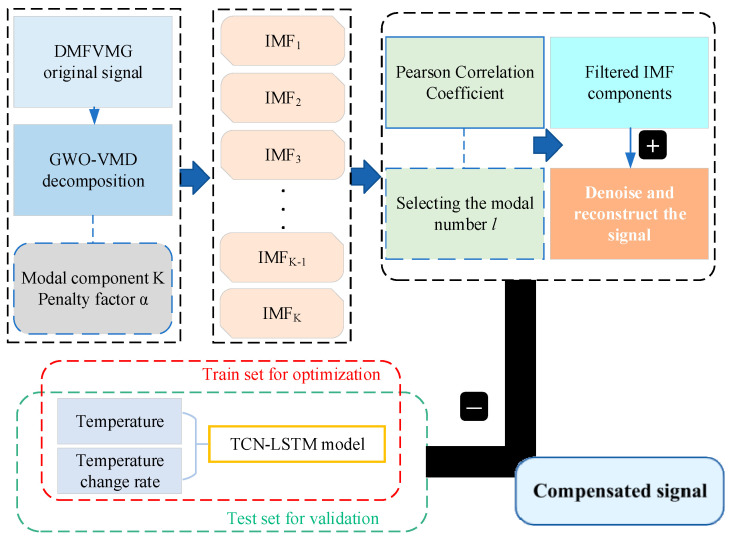
Schematic diagram of the gyroscope temperature compensation model.

Figure 5 depicts the schematic representation of the model for compensating the temperature-induced drift in gyroscopes. The process begins with the decomposition and subsequent reconstruction of the drift signal, employing the GWO-VMD technique. During this phase, the feature components of the signal are retained, while the noise components are eliminated, resulting in a purified signal. This cleaned signal is then input into the TCN-LSTM hybrid forecasting model to refine its predictive capabilities. The final step involves subtracting the model’s predicted output from the original gyroscope drift signal, yielding the compensation signal. Through these steps, the model for temperature compensation in gyroscopes is successfully constructed.

## 4. Experimental Test

**Figure 6:**
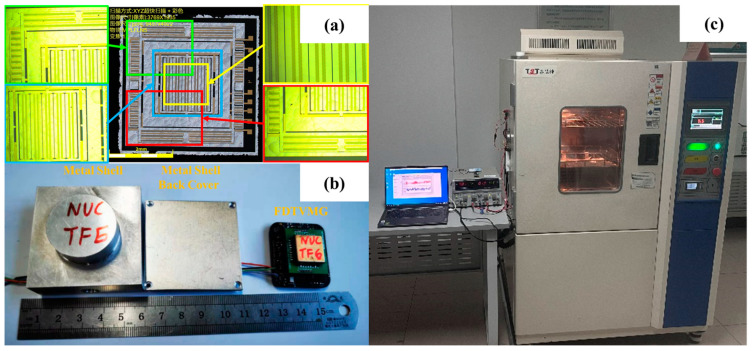
(**a**) Field emission scanning electron microscope (FESEM) image (color added). (**b**) DMFVMG prototype photo. (**c**) Experimental environment settings.

In order to validate the necessity of the GWO-VMD-based TCN-LSTM temperature compensation model, we conducted a temperature experiment to evaluate the temperature response characteristics of the DMFVMG. Figure 6 illustrates the experimental arrangement and the construction of the gyroscope. In our experiments, we employed a DMFVMG characterized by the specifications of ±300°/s for its range, −19.06 mV/°/s for the scale factor and an intercept of 95.43 mV. Additionally, a precision detection circuit was meticulously integrated into its internal PCB. The circuit interfaced with external components via three dedicated data lines. One of these lines facilitated the connection between the gyroscope’s weak signal interface and the chip architecture. The remaining two lines were designated for linking the gyroscope’s sensing and actuation circuits, respectively. To mitigate the effects of the ambient humidity on the gyroscope’s operational performance, the device had been encapsulated in rubber as a protective measure. In addition, given the lightweight nature of the DMFVMG and its sensitivity to shock and vibration, electrical tape was used to secure it to ensure stability during the experiment.

Over the course of this experiment, we utilized a laptop computer (Dell Inc., Round Rock, TX, USA), a multimeter (Agilent Technologies, Inc., Santa Clara, CA, USA), a power supply unit (GW Instek, Taipei, Taiwan) and a temperature-regulated oven (UNIQUE Industries, Osaka, Japan) as our primary equipment [27]. Initially, we powered the gyroscope for a duration of 90 min at ambient temperature. Concurrently, the temperature-regulated oven was set to cool to 40 °C and was allowed to stabilize at this temperature for 45 min.

**Figure 7:**
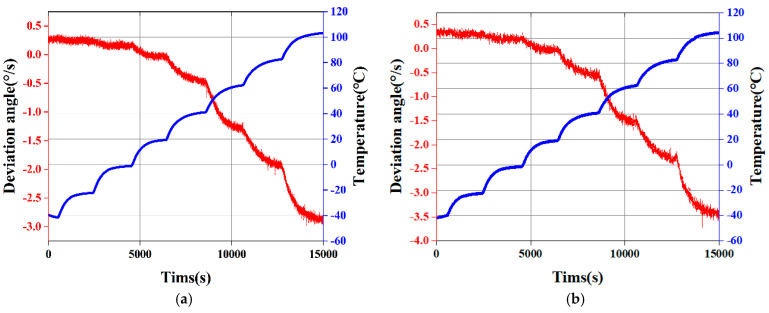
(**a**) Data from the training phase of the experiment. (**b**) Data from the testing phase of the experiment.

For our studies confirming the importance of a temperature-adjusted model, we evaluated the DMFVMG over a temperature spectrum extending from 40 °C to 100 °C, gathering data at 20 °C intervals. To ensure precise temperature synchronization between the gyroscope and the temperature control box, as well as to maintain the stability of the gyroscope’s drift signal, we allowed the gyroscope to remain at each fixed temperature point for approximately 30 min. Throughout this period, continuous data acquisition was performed to guarantee that the output data accurately represented the gyroscope’s performance across the various temperature conditions. To mitigate the impact of random errors on our experimental outcomes, we conducted five sets of temperature experiments. A comparative analysis revealed a high degree of consistency among the collected data. Consequently, we selected two of these datasets for further analysis and processing: one set for model training (as depicted in Figure 7a) and another for model testing (as shown in Figure 7b).

## 5. Experiment Analysis

### 5.1. Result

**Figure 8:**
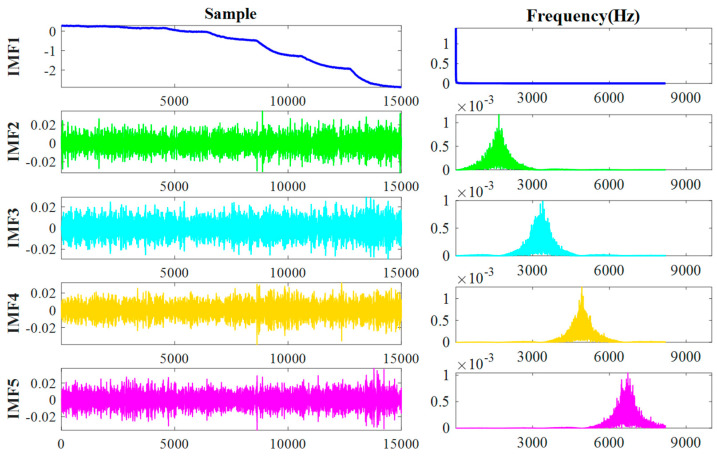
Decomposition results and spectrograms based on GWO-VMD.

The GWO-VMD method was implemented in the decomposition process of the gyroscope output signal, successfully breaking down the complex signal into seven intrinsic modal functions (IMF1 to IMF7). These IMF components represent different frequency bands of the signal: the low-frequency components predominantly reflect the temperature drift characteristics of the gyroscope, while the high-frequency components are primarily indicative of noise. As illustrated in Figure 8, the application of GWO-VMD enables us to distinctly identify and isolate the critical factors that influence measurement accuracy.

**Figure 9:**
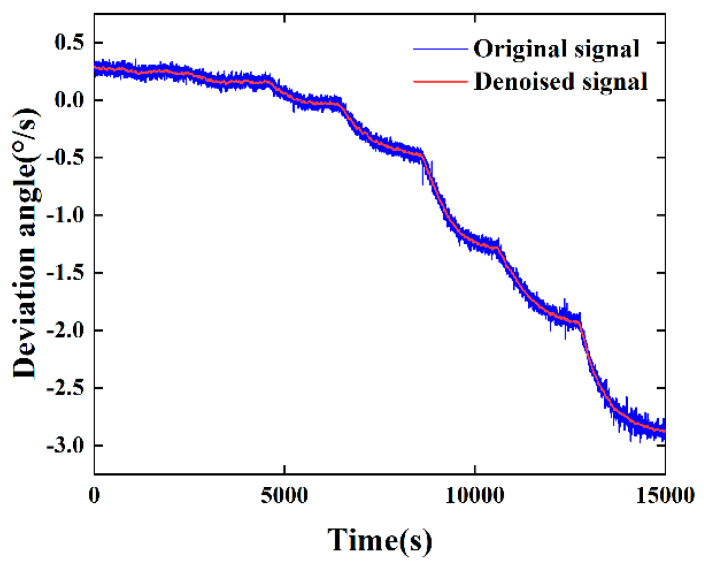
Comparison of DMFVMG signal before and after denoising.

In the subsequent processing of the Intrinsic Modal Functions (IMFs), our objective was to preserve the feature terms that accurately represent the true physical behavior of the gyroscope while effectively filtering out the noise terms that are intermingled with the signal. This critical step is essential for enhancing signal quality and diminishing the impact of errors. Ultimately, by reconstructing the filtered IMFs, we derived the denoised reconstructed signal. This denoising procedure not only markedly enhanced the signal’s clarity but also substantially boosted its resilience to external disturbances, such as temperature fluctuations. Visual comparisons before and after denoising, as depicted in Figure 9, corroborated the effectiveness and practicality of the method proposed in this paper.

Based on the temperature–output relationship observed in the gyroscope’s temperature experiments, it has been determined that the DMFVMG exhibits distinct trends at various temperature levels. This relationship between temperature and output is highly nonlinear and irregular, indicating that the output cannot be solely mapped from the temperature dimension. To address this, this paper introduced the rate of temperature change as an additional input variable. This established a dual-input single-output mapping relationship, which enhanced the precision of the compensation prediction.

**Figure 10:**
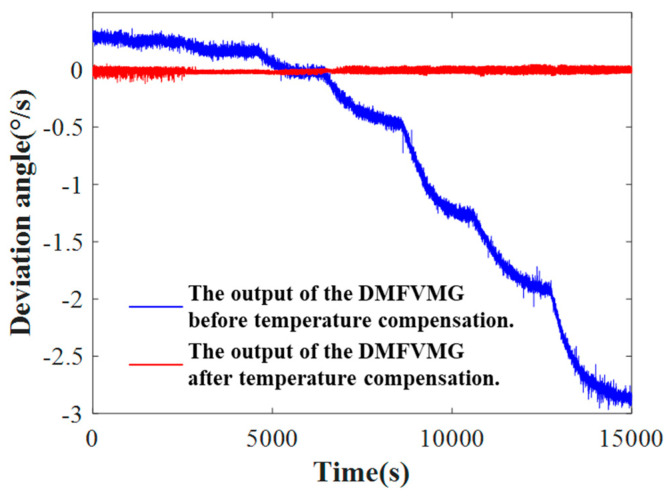
Comparison of effect after temperature compensation.

The two-dimensional temperature sequences and temperature feature terms constitute the training dataset for the compensation model. They are utilized to train the neural network and to develop a specific temperature-drift input-output mapping relationship tailored to the DMFVMG. After defining the training dataset, the TCN-LSTM neural network model undergoes training. Following this, the temperature and its rate of change from the test dataset are fed into the fine-tuned TCN-LSTM model to produce the predicted values for the test dataset. The original signals are then adjusted by subtracting these predicted values, yielding the output of the offline compensated signals as depicted in Figure 10. It is evident that temperature compensation is highly effective.

Furthermore, to demonstrate the effectiveness of the TCN-LSTM prediction model, we conducted a comparative analysis with several standalone predictive models, including the LSTM network, the TCN and the Backpropagation(BP) neural network and traditional polynomial fitting methods, specifically using a sixth-degree polynomial. To quantitatively evaluate the performance of the LSTM network, the TCN, the BP neural network, polynomial fitting and the TCN-LSTM models, we computed the prediction error for each model by comparing their predicted values with the actual values, as detailed in Table 2. The data in the table clearly indicates that the TCN-LSTM model outperformed the others in terms of prediction accuracy. This result underscores the advantages of our model for temperature compensation in gyroscopes.

### 5.2. Analysis

In this study, we employed the Allan ANOVA method to quantitatively assess both the raw signals from the gyroscope test setup and the output signals following compensation by the various algorithms. This analysis enabled us to extract the angular velocity random wander and zero-bias instability data for the original signal, as well as for the signals that underwent processing through the different compensation algorithms. The results are summarized in Table 3.

**Figure 11:**
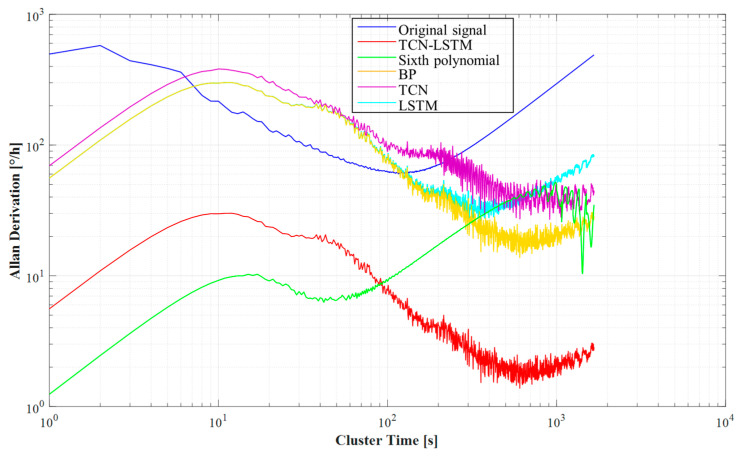
Graph of Allan ANOVA result.

The analysis results show in Figure 11 that the uncompensated raw signal had an angular velocity random wander of 102.929°/h/√Hz and a zero-bias instability of 63.70°/h. When we compensated the signal using the LSTM model, the angular velocity random wander of the signal was reduced to 39.2343°/h/√Hz and the zero-bias instability was reduced to 27.96°/h. Compensating the signal using the TCN model, the angular velocity random wander and zero-bias instability were obtained as 25.2652°/h/√Hz and 26.99°/h, respectively and they were further reduced to 22.6733°/h/√Hz and 13.79°/h after compensating with the BP neural network model. 19.7748°/h/√Hz and 6.27°/h were obtained for compensation with the sixth-degree polynomial model. Most notably, the angular velocity random wandering and zero-bias instability were significantly reduced to 17.6903°/h/√Hz and 1.38°/h, respectively, when we compensated with the TCN-LSTM model.

These results show that with the continuous optimization of the compensation algorithm, the angular rate random wander (ARRW) and zero-bias instability of the gyroscope were significantly improved, with the TCN-LSTM model performing particularly well in reducing these metrics.

## 6. Conclusions

In this study, to address the issue of temperature-induced drift in the output signal of a MEMS gyroscope, we proposed a TCN-LSTM temperature compensation model that incorporated GWO-VMD denoising. By employing GWO-VMD denoising, we could retain as much of the original signal’s useful information as possible while accurately extracting its relevant features. The TCN-LSTM model, compared to other traditional prediction algorithms, exhibited superior predictive performance, thereby enhancing the accuracy of the compensation model. The TCN-LSTM temperature compensation model, when combined with GWO-VMD denoising, could achieve a more precise compensation effect. The temperature experimental results indicate that the rate random wander of the output signal was reduced from 102.929°/h/√Hz to 17.6903°/h/√Hz and the bias instability was reduced from 63.70°/h to 1.38°/h. This represents a reduction of 82.81% for the rate random wander and 97.83% for the bias instability. These experimental results confirm the effectiveness and superiority of our proposed temperature compensation model [28,29,30].

## Figures and Tables

**Table 1:** Resonant frequencies associated with the modal behavior of the DMFVMG structure.

Mode Order	Resonant Frequency Value (Hz)	Remarks
1	10,094	Sense Mode
2	10,162	Drive Mode
3	14,986	z-axis vibration
4	15,661	Comb frame movement
5	15,811	Structure rotating around z-axis
6	16,181	Comb frame movement
7	18,234	Comb frame movement
8	18,242	Comb frame movement
9	18,251	Comb frame movement
10	18,254	Comb frame movement

**Table 2:** Predicted value error.

Model	Mean Absolute Error (MAE)	Mean Absolute Percentage Error (MAPE)	Root Mean Square Error (RMSE)
LSTM	0.0669	1.6561	0.1132
TCN	0.1293	0.8360	0.1854
BP	0.1597	1.4782	0.1977
Sixth polynomial	0.0567	0.7894	0.0859
TCN-LSTM	0.023	0.4391	0.0421

**Table 3:** Results of Allan ANOVA with different methods.

Model	ARRW (°/h/√Hz)	Zero-Bias Instability (°/h)
Original signal	102.929	63.70
LSTM	39.2343	27.96
TCN	25.2652	26.99
BP	22.6733	13.79
Sixth polynomial	19.7748	6.27
TCN-LSTM	17.6903	1.38

## Data Availability

The data used to support the findings of this study are available from the corresponding author upon request.
